# The “Real R0”: A Resection Margin Smaller Than 0.1 cm is Associated with a Poor Prognosis After Oncologic Esophagectomy

**DOI:** 10.1245/s10434-021-10121-y

**Published:** 2021-05-26

**Authors:** Penelope St-Amour, Michael Winiker, Christine Sempoux, François Fasquelle, Nicolas Demartines, Markus Schäfer, Styliani Mantziari

**Affiliations:** 1 Department of Visceral Surgery, University Hospital of Lausanne, Lausanne, Switzerland; 2 Institute of Pathology, University Hospital of Lausanne, Lausanne, Switzerland; 3 Faculty of Biology and Medicine, University of Lausanne (UNIL), Lausanne, Switzerland

## Abstract

**Background:**

Although resection margin (*R*) status is a widely used prognostic factor after esophagectomy, the definition of positive margins (*R*1) is not universal. The Royal College of Pathologists considers *R*1 resection to be a distance less than 0.1 cm, whereas the College of American Pathologists considers it to be a distance of 0.0 cm. This study assessed the predictive value of *R* status after oncologic esophagectomy, comparing survival and recurrence among patients with *R*0 resection (> 0.1-cm clearance), *R*0+ resection (≤ 0.1-cm clearance), and *R*1 resection (0.0-cm clearance).

**Methods:**

The study enrolled all eligible patients undergoing curative oncologic esophagectomy between 2012 and 2018. Clinicopathologic features, survival, and recurrence were compared for *R*0, *R*0+, and *R*1 patients. Categorical variables were compared with the chi-square or Fisher’s test, and continuous variables were compared with the analysis of variance (ANOVA) test, whereas the Kaplan-Meier method and Cox regression were used for survival analysis.

**Results:**

Among the 160 patients included in this study, 113 resections (70.6%) were *R*0, 34 (21.3%) were *R*0+, and 13 (8.1%) were *R*1. The *R*0 patients had a better overall survival (OS) and disease-free survival (DFS) than the *R*0+ and *R*1 patients. The *R*0+ resection offered a lower long-term recurrence risk than the *R*1 resection, and the *R* status was independently associated with DFS, but not OS, in the multivariate analysis. Both the *R*0+ and *R*1 patients had significantly more adverse histologic features (lymphovascular and perineural invasion) than the *R*0 patients and experienced more distant and locoregional recurrence.

**Conclusions:**

Although *R* status is an independent predictor of DFS after oncologic esophagectomy, the < 0.1-cm definition for *R*1 resection seems more appropriate than the 0.0-cm definition as an indicator of poor tumor biology, long-term recurrence, and survival.

**Supplementary Information:**

The online version contains supplementary material available at 10.1245/s10434-021-10121-y.

In recent years, the incidence of esophageal cancer, particularly adenocarcinoma, keeps rising in the Western world in relation to obesity and uncontrolled reflux disease.^1^,^2^ In parallel, improvements in neoadjuvant treatments yield a significant survival benefit for patients with locally advanced disease, related to the control of systemic spread as well as to the primary tumor’s downsizing and an increase in complete microscopic resection (*R*0) rates.^3^^-^6

Although resection margin status (*R*) is widely used as a prognostic factor after curative esophagectomy, published studies remain ambivalent concerning its actual predictive value. Several authors suggest that *R* status should be considered not as an independent prognosticator, but rather as part of a wider panel of unfavorable tumor biology markers such as lymphovascular invasion and depth of tumor infiltration.^7^^-^9

Currently, two different definitions of the *R* status are used in clinical practice. The Royal College of Pathologists (RCP) uses a more “strict” definition, considering *R*1 status as a clearance of < 0.1 cm,^10^ whereas the College of American Pathologists (CAP) defines a positive margin (*R*1) as a direct contact between the tumor and the surgical margin (clearance of 0.0 cm).^11^ Thus, the prognostic value of *R* status is subject to the different definitions used among studies, limiting comparability of surgical series.

Several additional reasons mandate a universally accepted definition of *R* status. It may be used as a surrogate marker of the oncologic quality of surgical resection,^12^ indicating better local control of the disease by neoadjuvant treatment,^13^ but it also can be considered as an indication to proceed to adjuvant treatment.^14^

This study aimed to assess the prognostic value of resection margin status (*R*) in terms of overall survival (OS) and recurrence after curative esophagectomy for cancer, and to compare the respective predictive values of the RCP and CAP definitions of *R*0.

## Methods

Our prospectively maintained institutional database provided all consecutive patients undergoing curative esophagectomy for cancer between January 2012 and December 2018 at the University Hospital of Lausanne, Switzerland. The inclusion criteria were age older than 18 years, squamous cell carcinoma (SCC) or adenocarcinoma (AC) of the esophagus or gastro-esophageal junction (Siewert I–II), and surgery with curative intent after discussion of each case by the multidisciplinary tumor board. Emergency surgery and all other histologic types were excluded from the analysis, as was the patient’s refusal to participate in clinical research. All the included patients provided an informed consent, and the study was approved by the Institutional Ethics Committee (protocol no. 2020-01175).

### Preoperative Workup, Treatment Strategy, and Outcome Measures

Preoperative evaluation consisted of oesogastroduodenoscopy (OGD) with endosonography (EUS) and biopsies, a thoraco-abdominal computed tomography (CT) scan, and a whole-body 18 F-fluorodeoxyglucose (FDG)-positron emission tomography (PET) scan. Locally advanced lesions of the distal esophagus mandated a diagnostic laparoscopy to exclude peritoneal implants.

According to international guidelines,^15^ early-stage tumors were treated with upfront surgery, whereas locally advanced tumors (cT3 and/or cN+) were treated with concurrent chemoradiation using fluorouracil (5-FU)/cisplatin or carboplatin/taxane at a dose of 41.4–50.4 Gy. In recent years, perioperative chemotherapy according to the 5-fluorouracile, leucovorin, oxaliplatin, and docetaxel (FLOT) regimen^4^ has been preferred for adenocarcinoma.

Severe postoperative complications were defined as Dindo-Clavien grade higher than 3a.^16^ Survival and recurrence were assessed in postoperative months, whereas early recurrence (ER) was defined as any site of tumor relapse during the first postoperative year.^17^ Patients who had postoperative (in-hospital) mortality were excluded from survival analysis.

Follow-up evaluation after surgery consisted of regular physical examination and imaging. According to in-hospital consensus, a CT scan was performed every 4 months during the first two postoperative years, then every 6 months for three additional years. In case of recurrence suspicion, the patient benefited from an endoscopy with histologic examination. Overall survival was defined as the delay between surgery and occurrence of death or the last follow-up visit if the patient was still alive, and disease-free survival (DFS) was determined by the date of recurrence or the last follow-up visit.

### Histopathologic Analysis: Tissue Handling

Tumor staging was defined by the seventh tumor-node-metastasis (TNM)/Union for International Cancer Control (UICC) staging system because none of the later modifications in the eighth version were of relevance to our study.^18^,^19^ The lymph-node ratio was defined as the ratio of positive-to-resected lymph nodes.

Surgical specimens were examined fresh after intraoperative orientation by the surgeon. Inking of the circumferential margin in two different colors (anterior and posterior) was performed. Then, the specimen was fixed unopened in formalin for 24–48 h. Tumor and tumor site were sampled and paraffin-embedded completely. If no macroscopic tumor was seen, the esophagus was completely embedded.

After embedding of the proximal and distal margins, serial sections from proximal to distal were sliced and examined carefully to sample all the areas of deepest tumor invasion and to establish the distance with the peripheral margins. Slides were stained with hematoxylin-eosin, and the *R* status was defined at the microscope with respect to the closest distance between the tumor and the surgical margin, circumferential or vertical (proximal or distal). The pathologist measured the distance to the circumferential resection margin (CRM) with an optical microscope using an ocular micrometer with a standardized stage scale.

All reports of histologic analyses were specifically reviewed for this study, and for equivocal reports, our senior pathologist (C.S.) reviewed the case for further clarification. Histologic response to treatment was defined with the Mandard tumor regression grade (TRG), in which TRG1 means a complete histologic response and TRG5 means no response at all.^20^

The patients were divided into three groups according to the closest microscopic clearance (circumferential or longitudinal) between the tumor and the resection margin as follows: *R*0 patients (> 0.1 cm of clearance), *R*1 patients (positive margins with 0.0 cm of clearance, CAP definition), and *R*0+ patients (margins with ≤ 0.1 cm of clearance, RCP definition).

For exploratory purposes, separate subgroup analyses were performed by histologic type (SCC or AC), as well as for patients who received neoadjuvant treatment (NAT+ subgroup). The analyses were intended to offer a better insight into the overall study results and to ascertain the absence of major bias according to histologic type. However, they were limited by the small number of patients per subgroup, not allowing separate and multivariate analyses.

### Statistical Analysis

Categorical variables were expressed as frequency (%) and compared with the chi-square or Fisher’s exact test, and continuous variables were expressed as median (interquartile range [IQR]) and compared with the analysis of variance (ANOVA) test. Time-to-event outcomes (survival and recurrence) were expressed as median (95% confidence interval [95% CI]). When median survival was not reached, the mean value for each group was used. The Kaplan-Meier method and the log-rank test were used for direct comparison of OS and DFS among the three groups, whereas Cox regression was used for uni- and multivariate survival analysis. Covariates with a *p* value lower than 0.10 in the univariate analysis were included in the multivariate model. Statistical analyses were performed with the *R* Studio (version 1.1. 383; Boston, MA, USA) and SPSS (version 23.0; Chicago, IL, USA) software.

## Results

### Baseline Demographics and Tumor Characteristics

**Fig. 1:**
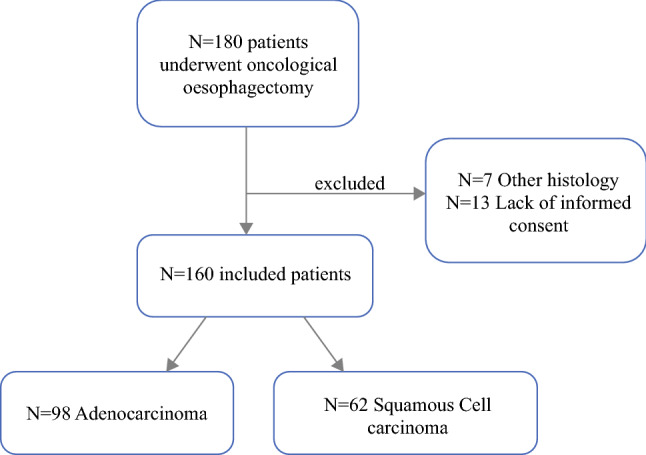
Flowchart of the study. Description of patients’ inclusion in the study group for analysis

Overall, 160 patients were eligible for the current study according to the inclusion criteria (Fig. 1). The study excluded 21 patients from the analysis (7 patients with other histology and 13 patients due to lack of informed consent). Among the included patients, 70.6% (*n* = 113) had an *R*0 resection, 21.3% (*n* = 34) had an *R*0+ resection, and 8.1% (*n* = 13) had an *R*1 resection. In 100% of the cases with a compromised resection margin (*R*0+ or *R*1), microscopic involvement was observed in the circumferential margin. As illustrated in Table 1, male gender was predominant in all groups, and the median age of the patients was 62 years. Tumor location was comparable among the three groups, whereas the predominant histologic type was adenocarcinoma.

**Table 1:** Demographic and preoperative characteristics of the study population according to their resection margin (*R*) status^a^

	All patients (*n* = 160) *n* (%)	*R*0 > 0.1 cm (*n* = 113) *n* (%)	R0+ ≤ 0.1 cm (*n* = 34) *n* (%)	R1 0 cm (*n* = 13) *n* (%)	*p* value
Male gender	127 (69.4)	86 (76.1)	30 (88.2)	11 (84.6)	0.274
ASA class					0.036
1–2	98 (61.3)	71 (62.8)	24 (70.6)	3 (23.1)	
3–4	62 (38.8)	42 (37.2)	10 (29.4)	10 (76.9)	
Median age: years (IQR)	62 (48-76)	62 (49-75)	70 (55-85)	59 (43-75)	0.019
Median BMI: kg/m^2^ (IQR)	24.5 (18.9-30.1)	24.3 (18.6-30.0)	25.3 (19.5-31.1)	25.7 (19.4-32.0)	0.634
Active smoking	59 (36.9)	45 (39.8)	10 (29.4)	4 (30.8)	0.486
Preoperative hiatal hernia	37 (23.1)	24 (21.2)	10 (29.4)	3 (23.1)	0.612
Tumor location					0.560
GEJ	50 (31.3)	32 (31.3)	12 (35.3)	6 (46.2)	
Distal third	67 (41.9)	49 (43.4)	14 (41.2)	4 (30.8)	
Middle third	40 (25.0)	30 (26.5)	7 (20.6)	3 (23.1)	
Upper third	2 (1.3)	2 (1.8)	0 (0)	0 (0)	
Histologic type					0.421
Squamous cell	62 (38.8)	46 (40.7)	10 (29.4)	6 (46.2)	
Adenocarcinoma	98 (61.2)	67 (59.3)	24 (70.6)	7 (53.8)	
cT stage					0.067
1	16 (10.0)	16 (14.2)	0 (0)	0 (0)	
2	22 (13.8)	17 (15.0)	4 (11.8)	1 (7.7)	
3	120 (75.0)	80 (70.8)	28 (82.4)	12 (92.3)	
4	2 (0.0)	0 (0)	2 (5.9)	0 (0)	
cN stage					0.736
0	59 (36.9)	43 (38.1)	12 (35.3)	4 (30.8)	
1	82 (51.3)	56 (49.6)	17 (50.0)	9 (69.2)	
2–3	16 (10.0)	11 (9.7)	5 (14.7)	0 (0)	
cM1 stage	6 (3.8)	4 (3.5)	2 (5.9)	0 (0)	0.623
Median SUVmax: g/l (IQR)	12.6 (9.5)	12.0 (11.4)	11.5 (8.3)	14.6 (7.1)	0.769
NAT	131 (81.9)	88 (77.9)	30 (88.2)	13 (100)	0.081
NAT type					0.219
Chemotherapy	24 (15.0)	13 (11.5)	6 (17.6)	5 (38.5)	
Chemoradiation	107 (66.9)	75 (66.4)	24 (70.6)	8 (61.5)	

*ASA*, American Society of Anesthesists score; *IQR*, interquartile range; *BMI*, body mass index; *GEJ*, gastroesophageal junction; *SUVmax*, baseline maximal standardized uptake value on PET-CT imaging; *NAT*, neoadjuvant treatment; *PET*, positron emission tomography; *CT*, computed tomography; *cTNM*, clinical tumor-node-metastasis

^a^cTNM stage is based on the 7th edition of Union for International Cancer Control (UICC) classification.^18^ Categorial variables are expressed as *n* (%), and continuous variables as median (IQR).

Preoperative staging showed cT3 tumors in most patients (70.8% of the *R*0 patients, 82.4% of the *R*0+ patients, and 92.3% of the *R*1 patients; *p* = 0.067), whereas the baseline cN stage was similar in all the groups. Six patients had oligometastatic (cM1) disease at diagnosis, but they were considered eligible for curative surgery after multidisciplinary discussion. Five of these patients had distant (supraclavicular or interaortocaval) lymph node invasion, which disappeared after neoadjuvant radiochemotherapy. The remaining patient had a human epidermal growth factor receptor 2-positive (HER2+) adenocarcinoma with a single liver metastasis and achieved a complete response to combined chemotherapy and immune therapy (trastuzumab). Neoadjuvant treatment (NAT) was administered to 81.9% of the patients (chemoradiation to 66.9%), without differences in treatment methods among the three groups (Table 1).

### Surgical Characteristics and Postoperative Outcomes

A thoraco-abdominal approach (Lewis procedure) with two-field lymphadenectomy was used in 83.1% of the cases, with no significant differences among the three groups. Minimally invasive surgery (laparoscopy) was used for the abdominal phase in 81.3% of the cases, and in both in the abdomen and thorax for 50% of the patients. Neither severe complications (35% of all the patients) nor anastomotic leakage rates (36.3% overall) in particular presented significant intergroup differences (online Appendix 1).

### Histopathologic Analysis (*Table **2*)

**Table 2:** Histologic examination of the surgical specimen for all the study population according to their resection margin (*R*) status^a^

	All patients (*n* = 160) *n* (%)	*R*0 > 0.1 cm (*n* = 113) *n* (%)	*R*0+ ≤ 0.1 cm (*n* = 34) *n* (%)	*R*1 0 cm (*n* = 13) *n* (%)	*p* value
pT stage					< 0.001
0	33 (20.6)	33 (28.9)	0 (0)	0 (0)	
1	40 (25.0)	38 (33.3)	2 (5.9)	0 (0)	
2	12 (7.5)	12 (10.5)	0 (0)	0 (0)	
3	72 (45.0)	30 (26.3)	31 (91.2)	11 (84.6)	
4	3 (1.9)	0 (0)	1 (2.9)	2 (15.4)	
pN stage					< 0.001
0	107 (66.9)	87 (76.3)	15 (44.1)	5 (38.5)	
1	30 (18.8)	17 (14.9)	9 (26.5)	4 (30.8)	
2	12 (7.5)	6 (5.3)	4 (11.8)	2 (15.4)	
3	10 (6.3)	2 (1.8)	6 (17.6)	2 (15.4)	
G					0.072
1	10 (6.3)	8 (7.0)	2 (5.9)	0 (0)	
2	43 (26.9)	30 (26.3)	10 (29.4)	3 (23.1)	
3	60 (37.5)	32 (28.1)	20 (58.8)	8 (61.5)	
L1 status	44 (27.5)	19 (16.7)	18 (52.9)	7 (53.8)	< 0.001
V1 status	37 (23.1)	14 (12.3)	15 (44.1)	8 (61.5)	< 0.001
Pn1 status	40 (25.0)	17 (14.9)	18 (52.9)	5 (38.5)	< 0.001
Median positive LN (IQR)	0 (0-1)	0 (0-6)	1 (0-4)	1 (0-5)	< 0.001
Median harvested LN (IQR)	21 (10-32)	21 (8-34)	21.5 (11.5-31.5)	24 (17-31)	0.772
Median LN ratio (IQR)	0 (0-0.05)	0 (0)	0.06 (0-0.22)	0.05 (0-0.18)	< 0.001
TRG					< 0.001
1–2	60 (37.5)	53 (46.5)	5 (14.7)	2 (15.4)	
3–5	66 (41.3)	29 (25.4)	26 (76.5)	11 (84.6)	

*G*, tumor differentiation grade; *L*1, lymphatic involvement; *V*1, vascular involvement; *Pn*1, perinervous involvement; *LN*, lymph node; *IQR*, interquartile range; TRG, tumor regression grade (Mandard);^20^ cTNM, clinical tumor-node-metastasis classification

^a^pTNM stage is based on the 7th edition of Union for International Cancer Control (UICC) classification.^18^ Categorical variables are expressed as *n* (%), and continuous variables as median (IQR).

The majority of the *R*0+ patients (94.1%) and *R*1 patients (100%) had full-thickness (ypT3-4) tumors compared with 26.3% in the *R*0 group (*p* < 0.001). The *R*0+ and *R*1 groups also presented with more lymphatic spread than the *R*0 group (pN+ in 55.9%, 61.6%, and 22%, respectively; *p* < 0.001). The *R*0+ and *R*1 patients had a trend of more poorly differentiated (G3) tumors (58.8% and 61.5%, respectively) than the *R*0 patients (28.1%) (*p* = 0.072). Overall, the *R*0+ and *R*1 patients presented significantly more adverse histologic features than the *R*0 patients (*p* < 0.001 for lymphatic [*L*], vascular [*V*], and perinervous [*Pn*] status). Finally, the histologic response to treatment (TRG) was significantly better in the *R*0 group, with 46.5% of the patients in this group achieving complete or excellent regression (TRG1-2) versus 14.7% in the *R*0+ group and 15.4% in the *R*1 group (*p* < 0.001).

### Tumor Recurrence Patterns

Locoregional (mediastinal) recurrence occurred in 16% (*n* = 18) of the *R*0 patients, 21.2% (*n* = 7) of the *R*0+ patients, and 58.3% (*n* = 7) of the *R*1 patients (*p* = 0.002), although anastomotic recurrence was similar in all the groups (4.5%, 0% and 1.3% respectively; *p* = 0.711). Distant recurrence occurred in 24.5% (*n* = 27) of the *R*0 patients, 45.5% (*n* = 15) of the *R*0+ patients, and 66.6% (*n* = 8) of the *R*1 patients (*p* = 0.002). Early tumor relapse within the first postoperative year occurred for 18.6% (*n* = 21) of the *R*0 patients, 44.1% (*n* = 15) of the *R*0+ patients, and 61.5% (*n* = 8) of the *R*1 patients (*p* < 0.001).

### R0 Status as a Predictor of OS and DFS

**Fig. 2:**
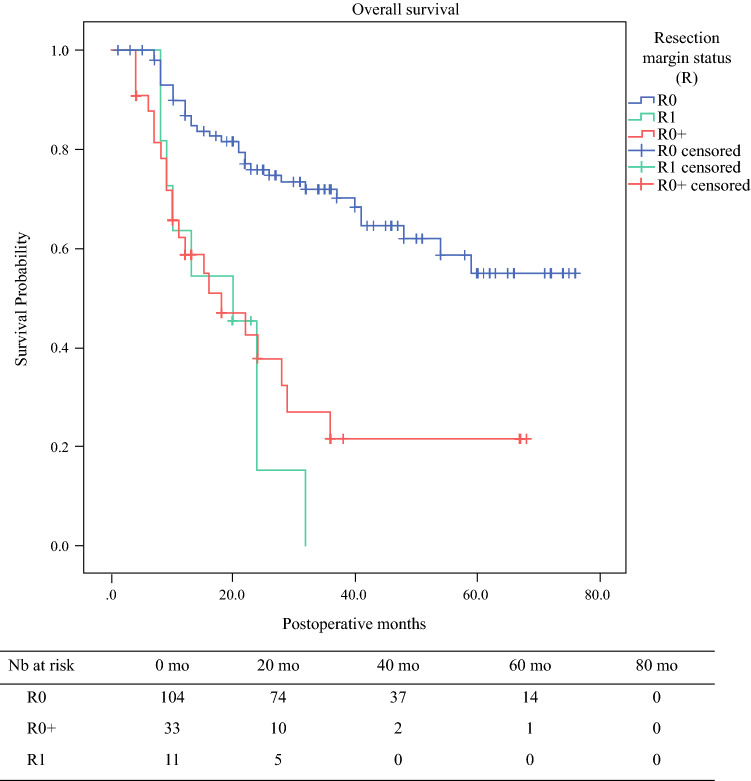
Overall survival (OS) for all the patients according to their *R* status^a^. mo, months; *R*0, resection margins > 1 mm; *R*0+, resection margins ≤ 1 mm; *R*1, resection margins 0 mm; CI, confidence interval. ^a^The *R*0 patients had a mean OS of 54.4 months (95% CI, 48.5–60.2 months), significantly better than the *R*0+ patients (median, 18 months (95% CI, 6.9–29.1 months; *p* < 0.001) and the *R*1 patients (median, 20 months; 95% CI, 11.5–28.5 months; *p* < 0.001). The difference between the *R*0+ and *R*1 patients was not significant (*p* = 0.558)

Of the 160 patients initially included in the study, 12 (7.5 %) were excluded from survival analysis due to in-hospital mortality. Among the remaining 148 patients, OS was significantly better for the *R*0 patients (mean, 54.4 months; median not reached; 95% CI, 48.5–60.2 months) than for the *R*0+ patients (median, 18 months; 95% CI, 6.9–29.1 months) or the *R*1 patients (median, 20 months; 95% CI, 11.5–28.5 months) (Fig. 2).

Pairwise comparisons showed a significant difference between the *R*0 and *R*0+ patients (*p* < 0.001) as well as between the *R*0 and *R*1 patients (*p* < 0.001), but not between the *R*0+ and *R*1 patients (*p* = 0.558). In the multivariate analysis, the *R*0+ patients presented a trend toward worse OS compared with the *R*0 patients (hazard ratio [HR], 2.22; 95% CI, 0.97–5.10; *p* = 0.059). The independent predictors of OS included initial cN status, baseline FDG-PET/CT maximum standardized uptake value (SUVmax) of the primary tumor, and the ratio of positive/resected lymph nodes (lymph node ratio) upon final pathology (Table 3).

**Table 3:** Cox regression analysis for overall survival (OS) of all patients^a^

	Unadjusted HR	95 % CI	*p* value	Adjusted HR	95 % CI	*p* value
Age	1.02	0.99–1.05	0.111			
Histology						
Squamous cell	1					
Adenocarcinoma	0.92	0.55–1.54	0.766			
cT stage						
cT1	1			1		
cT2	1.21	0.29–5.05	0.797	1.33	0.36–4.90	0.669
cT3-4	3.02	0.94–9.67	0.063	1.78	0.48–6.57	0.385
cN stage						
cN0				1		
cN1	1.83	1.04–3.23	0.037	2.62	1.60–9.07	0.029
cN2	1.22	0.44–3.33	0.701	4.57	0.47–1.02	0.053
cN3	1.23	0.16–9.23	0.838	9.04	0.01–7.95	0.312
cM stage	1.11	0.35–3.56	0.857			
SUVmax	1.04	1.01–1.07	0.004	1.09	1.04–1.14	< 0.001
NAT	3.96	1.44–10.9	0.008	3.22	0.05–209.8	0.583
Severe complications	1.05	0.60–1.84	0.857			
pT stage						
pT0	1			1		
pT1	0.31	0.10–1.01	0.051	1.35	0.24–7.44	0.731
pT2	0.53	0.12–2.44	0.417	0.52	0.07–3.83	0.525
pT3-4	1.02	1.39–5.51	0.004	0.93	0.19–4.40	0.928
LN ratio	37.28	11.52–120.6	< 0.001	20.15	3.59–112.9	< 0.001
TRG						
1–2	0.26	0.14–0.47	< 0.001	0.45	0.13–1.52	0.199
3–5	1					
V1 status	3.84	2.25–6.54	< 0.001	1.36	0.54–3.40	0.508
L1 status	3.66	2.18–6.16	< 0.001	1.13	0.47–2.75	0.778
Pn1 status	2.61	1.55–4.39	0.0003	1.08	0.51–2.26	0.846
R status						
* R*0	1			1		
*R*1	4.69	2.19–10.03	< 0.001	1.91	0.69–5.21	0.208
*R*0+	3.65	2.09–6.39	< 0.001	2.22	0.97–5.10	0.059

HR, hazard ratio; 95% CI, 95% confidence interval; *SUVmax*, maximal standardized uptake value on baseline (pre-treatment) FDG-PET/CT;* NAT*, neoadjuvant treatment; *LN*, lymph node; *L1*, lymphatic involvement; *V1*, vascular involvement; *Pn1*, perinervous involvement; *TRG*, tumor regression grade (Mandard); 20*FDG*, F-fluorodeoxyglucose; *PET*, positron emission tomography; *CT*, computed tomography

^a^Uni- and multivariate analyses of determinants of overall survival. Severe complications were defined according to Dindo-Clavien classification (≥ 3a).^16^

**Fig. 3:**
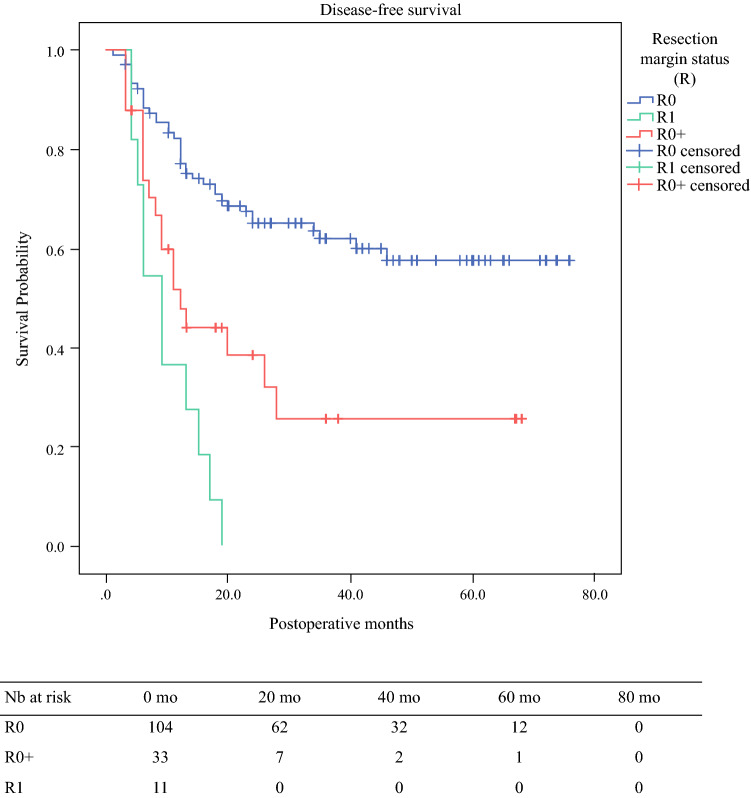
Disease-free survival (DFS) for all the patients according to their *R* status^a^. mo, months; *R*0, resection margins > 1 mm; *R*0+, resection margins ≤ 1 mm; *R*1, resection margins 0 mm; CI, confidence interval. ^a^The *R*0 patients had a better DFS (mean, 50.7 months; 95% CI, 44.3–57.0 months), than the *R*0+ patients (median, 12 months; 95% CI, 7.3–16.7 months; *p* = 0.001) and the *R*1 patients (median, 9 months; 95% CI, 5.9–12.1 months; *p* < 0.001). The difference between the *R*0+ and *R*1 patients also was significant (*p* = 0.027)

A significant DFS benefit was seen for the *R*0 patients (median not reached; mean, 50.7 months; 95% CI, 44.3–57.0 months) compared with the *R*0+ patients (median, 12 months; 95% CI, 7.3–16.7 months) and the R1 patients (median, 9 months; 95% CI, 5.9–12.1 months). Pairwise comparisons showed a significantly better DFS for the *R*0 patients than for the *R*0+ patients (*p* = 0.001), and for the *R*0 patients than for the *R*1 patients (*p* < 0.001), but also for the R0+ patients than for the *R*1 patients (*p* = 0.027) (Fig. 3). In the multivariate Cox regression, *R* status remained independently related to DFS, with the *R*1 patients having a significantly worse DFS than the *R*0 patients (HR, 2.91; 95% CI, 1.09–7, 74; *p* = 0.032). Other independent predictors of DFS were the primary tumor’s baseline FDG-PET/CT SUVmax, lymph-node ratio, and the Mandard regression grade (TRG) (Table 4).

**Table 4:** Cox regression analysis for disease-free survival (DFS) of all patients^a^

	Unadjusted HR	95 % CI	*p* value	Adjusted HR	95% CI	*p* value
Age	1.00	0.98–1.03	0.789			
Histology						
Squamous cell	1					
Adenocarcinoma	1.18	0.71–1.97	0.509			
cT stage						
cT1	1			1		
cT2	1.01	0.28–3.59	0.983	0.80	0.18–3.55	0.770
cT3	2.38	0.86–6.57	0.093	1.21	0.87–12.45	0.563
cN stage						
cN0	1			1		
cN1	2.01	1.14–3.54	0.015	0.51	0.22–1.17	0.111
cN2	2.28	0.98–5.29	0.055	0.78	0.20–3.00	0.723
cN3	1.17	0.15–8.79	0.879	0.24	0.02–22.3	0.536
cM stage	1.70	0.62–4.69	0.303			
SUVmax	1.03	1.00–1.058	0.014	1.05	1.01–1.09	0.012
NAT	3.07	1.32–7.09	0.009	4.25	0.05–374.9	0.526
Severe complications	0.70	0.39–1.25	0.229			
pT stage						
pT0	1			1		
pT1	0.33	0.13–0.88	0.027	0.39	0.06–2.56	0.329
pT2	0.36	0.08–1.60	0.181	0.12	0.01–1.00	0.051
pT3-4	2.05	1.11–3.81	0.022	0.21	0.04–1.20	0.079
LN ratio	69.88	21.11–231.3	< 0.001	70.84	10.9–458.8	< 0.001
TRG						
1–2	0.269	0.15–0.48	< 0.001	0.112	0.02–0.54	0.006
3–5	1					
V1 status	3.46	2.08–5.78	< 0.001	1.25	0.50–3.14	
L1 status	3.63	2.22–5.94	< 0.001	0.73	0.29–1.80	
Pn1 status	2.09	1.25–3.49	0.005	0.79	0.39–1.61	
R status						
*R*0	1			1		
*R*1	5.89	2.94–11.84	< 0.001	2.91	1.09–7.74	0.032
*R*0+	2.64	1.51–4.62	< 0.001	1.85	0.82–4.16	0.136

HR, hazard ratio, 95% CI, 95% confidence interval; *SUVmax*, maximal standardized uptake value on baseline (pre-treatment) FDG-PET/CT; *NAT*, neoadjuvant treatment; *LN*, lymph node; *TRG*, tumor regression grade (Mandard);^20^*V1*, vascular involvement; *L1*, lymphatic involvement; *Pn1*, perinervous involvement; *FDG*, F-fluorodeoxyglucose; *PET*, positron emission tomography; *CT*, computed tomography

^a^Uni- and multivariate analyses of determinants of disease-free survival. Severe complications were defined according to Dindo-Clavien classification (≥ 3a).^16^

### Subgroup Analyses by Histologic Subtype

To assess the potential impact of varied tumor biology according to histologic type, exploratory subgroup analyses were performed for the SCC and AC patients (online Appendices 2–5). The baseline demographics and tumor characteristics were similar in the two subgroups. Overall survival was comparable in the two subgroups, with longer OS for the *R*0 patients than for the *R*0+ and *R*1 patients and similar OS between the *R*0+ and *R*1 groups. The *R*0 patients had a better DFS than the two others in both the AC and SCC subgroups. Moreover, when comparing DFS between R0+ and R1 patients with SCC, we found better outcomes for R0+ patients (mean DFS 16.6 months, median not reached; 95% CI 10.1-23.2 for R0+, median DFS 5 months, 95% CI 3.0-6.9 months for R1, p=0.049).

### Subgroup Analyses, for Patients with NAT

In the subgroup analysis of the patients who received NAT (online Appendices 6 and 7), the baseline demographics and the tumor characteristics were comparable among the three groups and similar to those of the overall study population. Kaplan-Meier analyses showed significantly better OS and DFS for the *R*0 patients than for the two other groups, whereas the *R*0+ and *R*1 groups had similar outcomes.

## Discussion

In the current study, the patients with a margin status of ≤ 0.1 cm (*R*0+) or 0.0 cm (*R*1) had significantly worse long-term outcomes than those with a “real” *R*0 resection (> 0.1 cm). The *R*0+ and *R*1 patients demonstrated a higher incidence of additional histologic characteristics associated with poor prognosis, such as extensive lymph node spread, lymphovascular and perineural invasion, and poor response to neoadjuvant treatment. Finally, *R* status itself was an independent predictor of DFS, but not OS.

In the literature, the rates of *R*1 resection after esophagectomy present a wide variation, depending on the definition used. In the current study, the *R*1 rate was 21.2% with the RCP criteria and 8.1% with the CAP criteria. These rates are substantially lower than in the meta-analysis published by Chan et al.^21^ in which 36.5% and 15.3% of patients had *R*1 resections with the respective definitions.

When the RCP criteria are used, *R*1 resection rates as high as 41.3% have been reported,^22^ reaching 67.4% for full-thickness tumors (T3).^23^ It is thus clear that variable definitions of *R*1 resection after esophagectomy represent a limitation to outcome comparison among published series. However, the appropriate *R*0 definition, and inversely, the *R*1 definition, is more than a question of scientific rigor. Frequently, *R*0 rates are used in the surgical literature as a surrogate marker for the quality of oncologic resection. Schlick et al.^12^ reported that surgical technique, hospital volume, and surgeon expertise can influence R0 rates, suggesting positive margins as an indicator of technical failure or inexperience.

However, caution is needed before surgical quality is interpreted through R0 resection rates alone. Except for the obvious drawback of variable (RCP and CAP) definitions, which are not always clearly stated, other factors are known to influence locoregional disease control in modern esophageal cancer management. For instance, neoadjuvant treatment has greatly improved the prognosis of patients through systemic disease control, but also through local tumor downsizing. Von Dobeln et al. 24 in the NeoRes trial showed higher R0 resection rates for locally advanced lesions when chemoradiation was used instead of chemotherapy alone.

In the current analysis, neither the type of surgical approach nor the NAT presented significant differences among the *R*0, *R*0+, and *R*1 patients. Among the baseline demographics, American Society of Anesthesiology (ASA) classes 3 and 4 were more prevalent in the R1 group in the current series. Although this difference reached statistical significance, we cannot assume a pathophysiologically plausible relationship between ASA class and radicality of tumor resection.

Interestingly, the mean age in the *R*1 group actually was younger, but all the other baseline demographics and staging features were similar among the three groups. Thus, we cannot conclude that *R*1 patients have a worse comorbid status leading to a survival bias against them.

Furthermore, although histologic types (AC and SCC) were comparable among the three *R* groups, separate exploratory subgroup analyses were performed. The outcome for each histologic type remained similar to that for the overall cohort, with the exception of DFS in the SCC subgroup, which was significantly better for the R0+ patients than for the R1 patients. Interestingly, the well-known superiority of radio-chemosensibility for SCC compared with AC did not seem to have an impact on the *R*0 resection rates or other adverse histologic features. However, the three groups had other notable differences in tumor biology and aggressiveness.

As other recent studies suggest, *R* status is an indicator of tumor biology in esophageal cancer, together with several other histologic features such as lymphatic (*L*), vascular (*V*), perineural invasion (*Pn*), and response to systemic treatment.^9^,^25^–^31^ Depypere et al.^25^ in a series of pT3 lesions confirmed that a poor response to neoadjuvant treatment and extensive lymph node involvement were significantly higher among *R*0+ and *R*1 patients than among *R*0 patients. Similarly, in our study the *R*0+ and *R*1 patients had significantly higher rates of locoregional lymphatic spread, vascular and perineural invasion, and poor response to neoadjuvant treatment than the *R*0 patients. Thus, *R* status should be considered in the broader context of the tumor’s biologic behavior.

Although previous authors have reported a limited prognostic value of *R* status when other biologic parameters are accounted for,^22^,^26^,^27^ the current analysis showed an independent correlation with DFS and a trend to significance for OS, highlighting the importance of radical negative-margin oncologic resection even in the presence of adverse histologic features.

A recent meta-analysis of 2433 esophageal cancer patients^21^ evaluated long-term survival and recurrence in relation to their *R* status assessed with the RCP and CAP definitions. The *R*0+ patients (RCP criteria) had an HR of 2.52 (*p* < 0.001) and the *R*1 patients (CAP criteria) an HR of 4.02 (*p* < 0.001) for long-term overall mortality compared with the *R*0 patients. Thus, although the CAP criteria may define a higher-risk group, both 0.0 cm and < 0.1 cm project an inferior prognosis for *R*0 and should be considered as *R*1.

More recent data confirm these findings, indicating the RCP definition as more appropriate,^22^,^26^,^27^ whereas Knight et al.^22^ suggested an even larger resection margin (< 0.2 cm) for a better relation to survival. Similar data have been reported for rectal cancer, in which a > 0.1-cm circumferential resection margin has been correlated with lower locoregional recurrence rates.^32^,^33^

In the current study, the > 0.1-cm margin also is supported because both the *R*0+ and *R*1 patients had a clearly worse OS and DFS than the *R*0 patients. The *R*0+ and *R*1 patients did not differ in terms of OS, but the *R*0+ patients had a longer DFS than the *R*1 patients (median DFS, 12 vs. 9 months; *p* = 0.027). Thus, although an increasingly worse outcome is observed from *R*0 to *R*0+ to *R*1 status, both *R*0+ and *R*1 patients have a higher risk of long-term mortality and recurrence than *R*0 patients. In this context, it seems inappropriate to associate *R*0+ with negative margins because this would be falsely reassuring and may deprive several patients of the benefits offered by adjuvant treatment.

Indeed, in addition to its prognostic value for long-term outcomes, *R* status is used in the decision-making process of esophageal cancer management. Often, *R*1 resection is an argument for adjuvant treatment during multidisciplinary tumor board discussions because it has been suggested that radiotherapy in particular offers a survival benefit.^14^,^26^ The current analysis showed more locoregional but also more distant metastatic recurrences for the *R*0+ and *R*1 patients than for the *R*0 patients, although anastomotic recurrence remained a rare event in all three groups. Thus, we suggest that after an <0.1-cm (*R*1) resection, adjuvant treatment should be targeted not only against the residual microscopic locoregional disease, but also against the systemic micro-metastases responsible for distant spread.

Our study had some limitations. First, its relatively small sample did not allow a separate analysis for the AC and SCC subgroups. However, because no differences were observed in histologic type or NAT method among the three *R* groups, this inherent difference in radio-chemosensibility between SCC and AC did not seem to introduce a significant bias.

Missing data in the histologic analysis represent another drawback. Despite the specific search for the data by thoroughly reviewing pathology reports, tissue samples were not re-analyzed to complete missing parameters. This issue has been reduced significantly in recent years as pathology reports have become more systematic and exhaustive. In addition, because the majority of the patients received neoadjuvant chemoradiation, adjuvant treatment was very rarely used in this series, not allowing a specific analysis of its impact in case of *R*0+ or *R*1 resection.

These limitations are counterbalanced by a recent and homogeneous series, a meticulous systematic handling of surgical samples during histologic analysis, a precise review of histology reports specifically for this study, and both rigorous methodology and statistical analysis.

In conclusion, because both definitions of positive resection margins are associated with a worse prognosis, the broader RCP definition (< 0.1 cm) for *R*1 resection seems to be of a higher sensitivity than the CAP definition (0.0 cm) as an indicator of poor tumor biology, adverse long-term recurrence, and survival for patients undergoing oncologic esophagectomy.

## Electronic supplementary material

Below is the link to the electronic supplementary material.

Supplementary file 1 (PDF 482kb)
