# Supplementary material for: The “Real R0”: A Resection Margin Smaller Than 0.1 cm is Associated with a Poor Prognosis After Oncologic Esophagectomy
Source: Ann Surg Oncol. 2021 May 26;28(12):7095–106. doi: 10.1245/s10434-021-10121-y (PMC8519834; doi:10.1245/s10434-021-10121-y)
Supplement: Supplementary file 1 — (PDF 482kb) [file 10434_2021_10121_MOESM1_ESM.pdf]

Online appendix 1 Surgical technique and post-operative course for all the study population, according to their R status.

	**All patients N=160**	**R0 >0.1cm N=113**	**R0+ ≤0.1cm N=34**	**R1 0cm N=13**	*P-value*
Type of resection					0.157
Lewis Santy	133 (83.1)	94 (83.2)	9 (69.2)	29 (85.3)	
Mckeown	19 (11.9)	15 (13.3)	2 (15.4)	2 (5.9)	
Transhiatal	9 (5.6)	4 (3.5)	2 (15.4)	3 (8.8)	
Minimally invasive approach					
Laparoscopy	130 (81.3)	91 (80.5)	10 (76.9)	29 (85.3)	0.858
Thoracoscopy	80 (50.0)	58 (51.3)	5 (38.5)	17 (50.0)	0.680
Severe complication	56 (35.0)	39 (34.5)	4 (30.8)	13 (38.2)	0.874
Anastomotic leakage	58 (36.3)	41 (36.3)	4 (30.8)	13 (38.2)	0.893

Categorial variables are expressed as N (%).

For Peer Review

Online appendix 2 Subgroup analysis for **squamous cell carcinoma patients (SCC)**

	**All patients N=62**	**R0 >0.1cm N=46**	**R0+ ≤0.1cm N=10**	**R1 0cm N=6**	*P-value*
Male gender	45 (72.6)	31 (67.4)	10 (100)	4 (66.7)	0.105
Age, years	64.1 [8.7]	63 [8]	70 [8.6]	62.7 [11.7]	0.066
ASA class					0.436
1-2	36 (58.1)	28 (60.9)	7 (70)	1 (16.7)	
3-4	26 (41.9)	18 (39.1)	3 (30)	5 (83.3)	
SUVmax (g/l)	13.9[9.6]	13.3 [8.9]	14.7 [10.8]	17.3 [13.5]	0.679
cT stage					0.324
1	7 (11.3)	7 (15.2)	0 (0)	0 (0)	
2	11 (17.7)	9 (19.6)	2 (20)	0 (0)	
3	44 (71)	30 (65.2)	8 (80)	6 (100)	
cN stage					0.770
0	27 (43.5)	21 (45.7)	3 (30)	3 (50)	
1	28 (45.2)	19 (41.3)	6 (60)	3 (50)	
2	6 (9.7)	5 (10.9)	1 (10)	0 (0)	
NAT	50 (80.6)	36 (78.3)	8 (80)	6 (100)	0.447
NAT type					0.541
Chemotherapy	2 (3.2)	1 (2.2)	0 (0)	1 (16.7)	
Chemoradiation	47 (75.8)	34 (73.9)	8 (80)	5 (83.3)	
Surgical approach					0.867
Lewis	41 (66.1)	31 (67.4)	7 (70)	3 (50)	
McKeown	16 (25.8)	12 (26.1)	2 (20)	2 (33.3)	
Transhiatal	5 (8.1)	3 (6.5)	1 (10)	1 (16.7)	
Severe complications	23 (37.1)	17 (37)	2 (20)	4 (66.7)	0.964
Anastomotic leakage	27 (43.5)	19 (41.3)	5 (50)	3 (50)	0.833
pT stage					<0.001
0	22 (35.5)	22 (47.9)	0 (0)	0 (0)	
1	16 (25.8)	15 (32.6)	1 (10)	0 (0)	

For Peer Review

2	3 (4.8)	3 (6.5)	0 (0)	0 (0)	
3	21 (33.9)	6 (13)	9 (90)	6 (100)	
pN stage					0.003
0	48 (77.4)	37 (80.4)	7 (70)	4 (66.7)	
1	10 (16.1)	8 (17.4)	1 (10)	1 (16.7)	
2	1 (1.6)	0 (0)	0 (0)	1 (16.7)	
3	2 (3.2)	0 (0)	2 (20)	0 (0)	
TRG					0.001
1-2	27 (43.5)	24 (52.2)	2 (20)	1 (16.7)	
3-5	17 (27.4)	6 (13)	6 (60)	5 (83.3)	
Early recurrence	16 (25.8)	7 (15.2)	4 (40)	5 (83.3)	0.001
Locoregional recurrence	16 (25.8)	10 (21.7)	2 (20)	4 (66.7)	0.059
Distant recurrence	14 (22.6)	8 (17.4)	3 (30)	3 (50)	0.179

Categorical variables are expressed as N (%), and continuous variables as median [IQR].

For Peer Review

Online appendix 3 Survival analysis for **squamous cell carcinoma (SCC)**

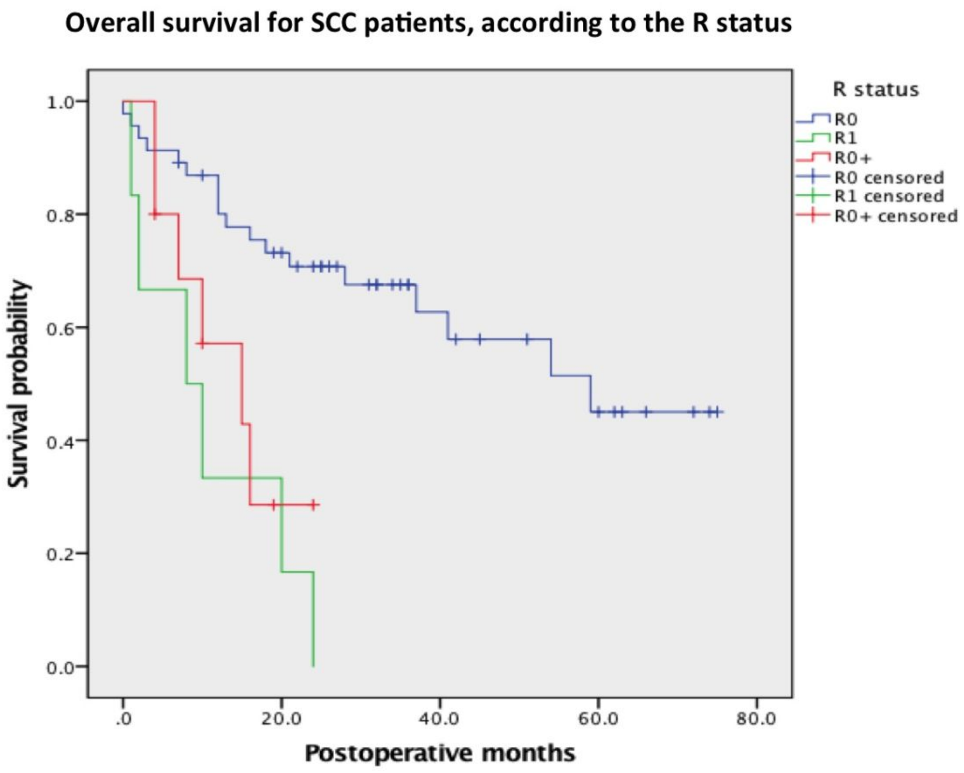

Nb at risk	0 mo	20 mo	40 mo	60 mo	80 mo
R0	45	30	13	6	0
R0+	9	1	0	0	0
R1	5	1	0	0	0

Figure legend: R0 patients had a mean OS of 48.6 months (95%CI 39.4-57.7), significantly better than R0+ (median 15 months 95%CI 3.0-26.9, p=0.012) and R1 patients (median 8 months, 95%CI 0-17.6, p<0.001). The difference was not significant between R0+ and R1 patients (p=0.391).

R0: resection margins >1mm R0+: resection margins ≤1mm R1: resection margins 0mm

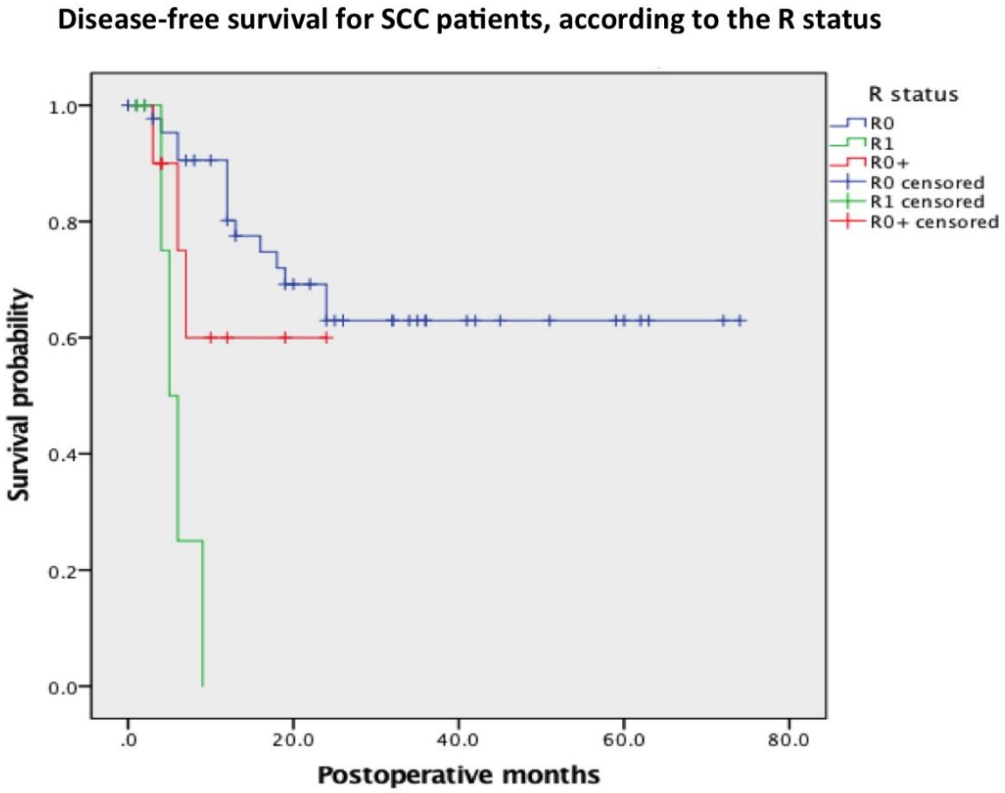

Nb at risk	0 mo	20 mo	40 mo	60 mo	80 mo
R0	45	23	9	4	0
R0+	9	1	0	0	0
R1	5	0	0	0	0

Figure legend: R0 patients had a mean DFS of 51.6 months (95%CI 42.1-61.0), not significantly different from R0+ (mean 16.6 months 95%CI 10.1-23.2, p=0.298) and significantly better than R1 patients (median 5 months, 95%CI 3.0-6.9, p<0.001). R0+ patients had a significantly better DFS than R1 (p=0.049).

R0: resection margins >1mm R0+: resection margins ≤1mm R1: resection margins 0mm

Review

Online appendix 4 Subgroup analysis for **adenocarcinoma patients (AC)**

	**All patients N=98**	**R0 >0.1cm N=67**	**R0+ ≤0.1cm N=24**	**R1 0cm N=7**	*P-value*
Male gender	82 (83.7)	55 (82.1)	20 (83.3)	7 (100)	0.475
Age, years	61.5[10.2]	60.4 [9.4]	65.3 [11.7]	59 [10.0]	0.101
ASA class					0.281
1-2	62 (63.3)	43 (64.2)	17 (70.8)	2 (28.6)	
3-4	36 (36.7)	24 (35.8)	7 (29.2)	5 (71.4)	
SUVmax (g/l)	13.8[8.8]	14.5 [10]	12.3 [5.8]	13.4 [4.7]	0.581
cT stage					0.276
1	9 (9.2)	9 (13.4)	0 (0)	0 (0)	
2	11 (11.2)	8 (12)	2 (8.3)	1 (14.3)	
3	78 (79.6)	50 (74.6)	22 (91.7)	6 (85.7)	
cN stage					0.637
0	32 (32.7)	22 (32.8)	9 (37.5)	1 (14.3)	
1	54 (55.1)	37 (55.2)	11 (45.8)	6 (85.7)	
2-3	10 (10.2)	6 (9)	4 (16.7)	0 (0)	
NAT	84 (85.7)	55 (82.1)	22 (91.7)	7 (100)	0.134
NAT type					0.138
Chemotherapy	22 (22.4)	12 (17.9)	6 (25)	4 (57.1)	
Chemoradiation	59 (60.2)	40 (59.7)	16 (66.7)	3 (42.9)	
Surgical approach					0.250
Lewis	91 (92.8)	63 (94)	22 (91.7)	6 (85.7)	
McKeown	3 (3.1)	3 (4.5)	0 (0)	0 (0)	
Transhiatal	4 (4.1)	1 (1.5)	2 (8.3)	1 (14.3)	
Severe complications	33 (33.7)	22 (32.8)	9 (37.5)	2 (28.6)	0.878
Anastomotic leakage	31 (31.6)	22 (32.8)	8 (33.3)	1 (14.3)	0.591
pT stage					<0.001
0	11 (11.2)	11 (16.5)	0 (0)	0 (0)	

For Peer Review

1	24 (24.5)	23 (34.3)	1 (4.2)	0 (0)	
2	9 (9.2)	9 (13.4)	0 (0)	0 (0)	
3	54 (55.1)	24 (35.8)	23 (95.8)	7 (100)	
pN stage					0.001
0	59 (60.2)	50 (74.6)	8 (33.3)	1 (14.3)	
1	20 (20.4)	9 (13.4)	8 (33.3)	3 (42.9)	
2	11 (11.2)	6 (9)	4 (16.7)	1 (14.3)	
3	8 (8.2)	2 (3)	4 (16.7)	2 (28.6)	
TRG					0.001
1-2	49 (50)	23 (34.3)	20 (83.3)	6 (85.6)	
3-5	33 (33.7)	29 (43.2)	3 (12.5)	1 (14.3)	
Early recurrence	28 (28.6)	14 (20.9)	11 (45.8)	3 (42.9)	0.046
Locoregional recurrence	16 (16.3)	8 (11.9)	5 (20.8)	3 (42.9)	0.043
Distant recurrence	36 (36.7)	19 (28.4)	12 (50)	5 (71.4)	0.010

Categorical variables are expressed as N (%), and continuous variables as median [IQR].

For Peer Review

Online appendix 5 Subgroup survival analysis for **adenocarcinoma (AC)**

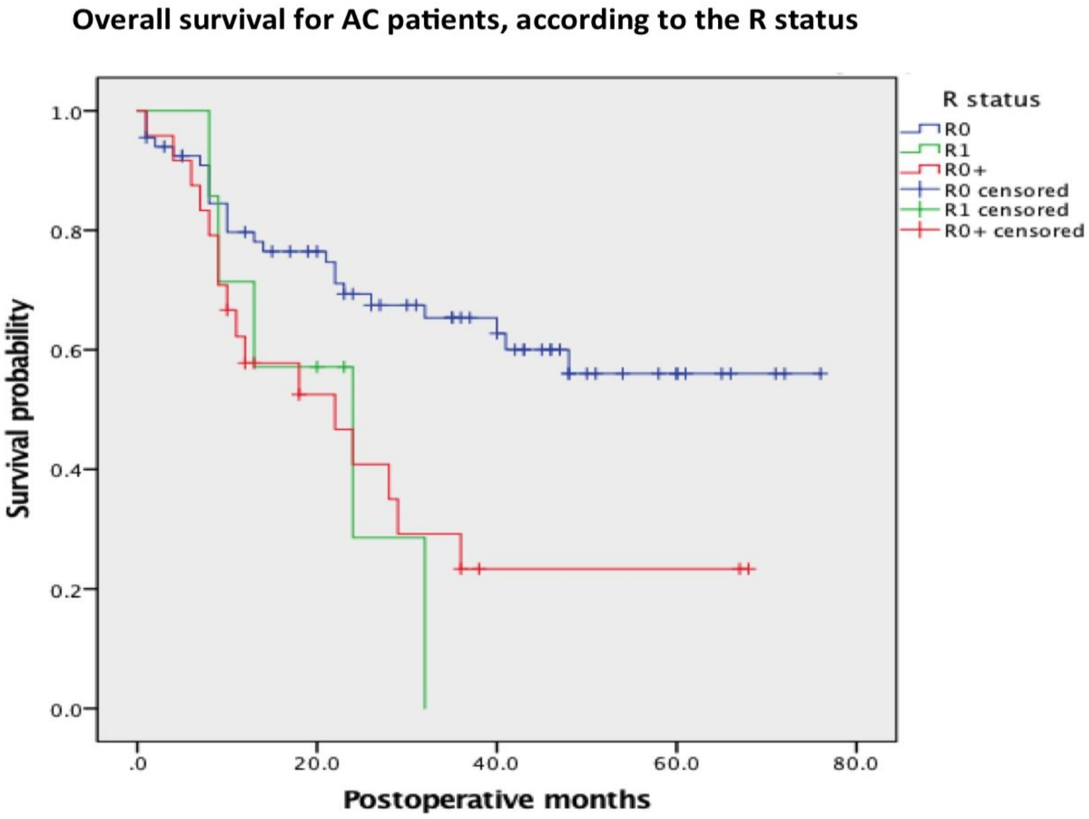

Nb at risk	0 mo	20 mo	40 mo	60 mo	80 mo
R0	66	43	27	7	0
R0+	23	4	0	0	0
R1	6	3	0	0	0

Figure legend: R0 patients had a mean OS of 51.1 months (95%CI 43.2-58.9), significantly better than R0+ (median 22 months 95%CI 7.2-40.8, p=0.004) and R1 patients (median 24 months, 95%CI 2.7-23.3, p=0.041). The difference was not significant between R0+ and R1 patients (p=0.818).

R0: resection margins >1mm R0+: resection margins ≤1mm R1: resection margins 0mm

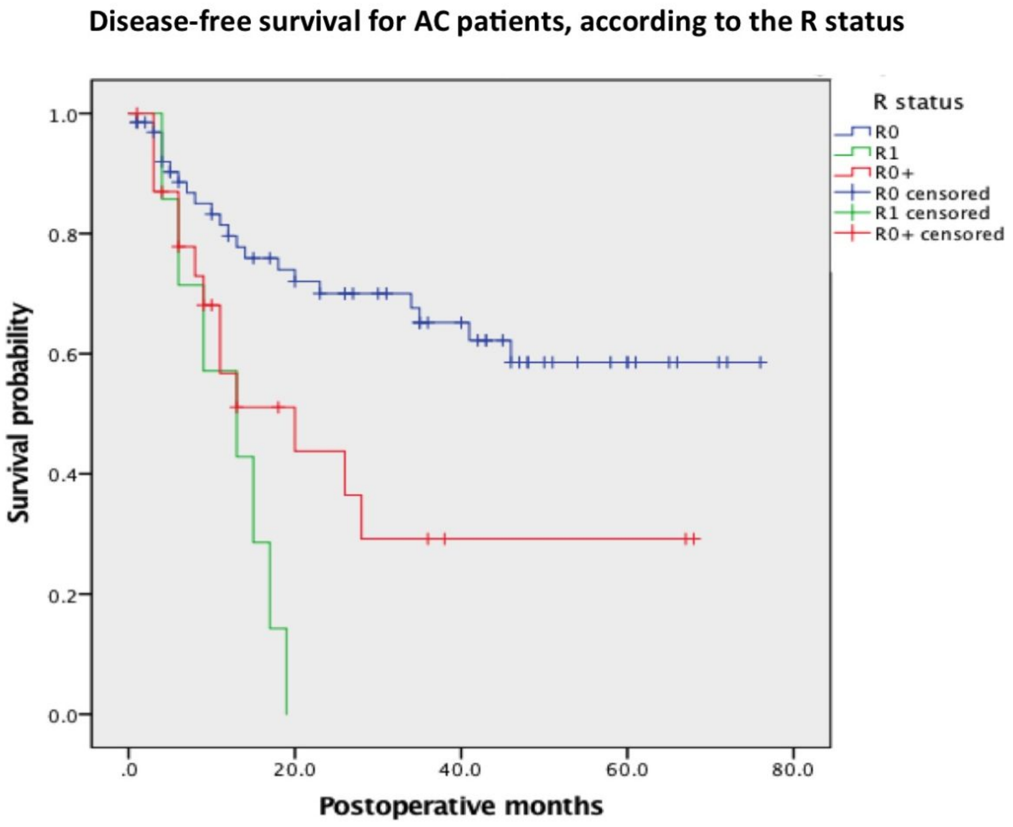

Nb at risk	0 mo	20 mo	40 mo	60 mo	80 mo
R0	65	37	22	6	0
R0+	23	6	0	0	0
R1	6	0	0	0	0

Figure legend: R0 patients had a mean DFS of 52.1 months (95%CI 43.9-60.2), significantly better then R0+ (median 20 months 95%CI 3.9-36.1, p=0.010) and R1 patients (median 13 months, 95%CI 2.7-23.3, p<0.001). The difference was not significant between R0+ and R1 patients (p=0.088).

R0: resection margins >1mm R0+: resection margins ≤1mm R1: resection margins 0mm

Review

Online appendix 6 Subgroup descriptive analysis for patients **who received NAT (SCC and AC)**

	**All patients N=131**	**R0 >0.1cm N=88**	**R0+ ≤0.1cm N=30**	**R1 0cm N=13**	*P-value*
Male gender	103 (78.6)	66 (75)	26 (86.7)	11 (84.6)	0.346
Age, years	62.7[9.7]	61.8 [8.8]	66.2 [11.4]	60.7 [10.5]	0.075
ASA class					0.041
1-2	81 (61.8)	57 (46.8)	21 (70)	3 (23.1)	
3-4	50 (38.2)	31 (35.2)	9 (30)	10 (76.9)	
SUVmax (g/l)	15.4 [9]	16.4 [9.5]	12.9 [7.2]	15 [9.1]	0.228
cT stage					0.653
1	4 (3.1)	4 (4.5)	0 (0)	0 (0)	
2	7 (5.3)	5 (5.7)	1 (3.3)	1 (7.7)	
3	120 (91.6)	79 (89.8)	29 (96.7)	12 (92.3)	
cN stage					0.749
0	31 (23.7)	18 (20.5)	9 (30)	4 (30.8)	
1	82 (62.6)	56 (63.6)	17 (56.7)	9 (69.2)	
2-3	15 (11,5)	11 (12.5)	4 (13.3)	0 (0)	
NAT type					0.316
Chemotherapy	24 (18.3)	13 (14.8)	6 (20)	5 (38.5)	
Chemoradiation	106 (80.9)	74 (84.1)	24 (80)	8 (61.5)	
Surgical approach					0.200
Lewis	109 (83.2)	74 (84.1)	26 (86.7)	9 (69.2)	
McKeown	16 (12.2)	12 (13.6)	2 (6.7)	2 (15.4)	
Transhiatal	6 (4.6)	2 (2.3)	2 (6.7)	2 (15.4)	
Severe complications	41 (31.3)	25 (28.4)	12 (40)	4 (30.8)	0.497
Anastomotic leakage	47 (35.9)	32 (36.4)	11 (36.7)	4 (30.8)	0.921
pT stage					0.001
0	30 (22.9)	30 (34.1)	0 (0)	0 (0)	
1	21 (16)	20 (22.7)	1 (3.3)	0 (0)	
2	9 (6.9)	9 (10.2)	0 (0)	0 (0)	

For Peer Review

3	71 (54.2)	29 (33)	29 (96.7)	13 (100)	
pN stage					0.004
0	84 (64.1)	66 (75)	13 (43.3)	5 (38.5)	
1	28 (21.4)	15 (17)	9 (30)	4 (30.8)	
2	9 (6.9)	5 (5.7)	2 (6.7)	2 (15.4)	
3	10 (7.6)	2 (2.3)	6 (20)	2 (15.4)	
TRG					0.001
1-2	65 (49.6)	29 (33)	25 (83.3)	11 (84.6)	
3-5	59 (45)	52 (59.1)	5 (16.7)	2 (15.4)	
Early recurrence	42 (32.1)	19 (21.6)	15 (50)	8 (61.5)	0.001
Locoregional recurrence	30 (22.9)	16 (18.2)	7 (23.3)	7 (53.8)	0.009
Distant recurrence	45 (34.4)	24 (27.3)	13 (43.3)	8 (61.5)	0.017

Categorical variables are expressed as N (%), and continuous variables as median (IQR).

For Peer Review

Online appendix 7 Subgroup survival analysis for patients **who received NAT (SCC and AC)**

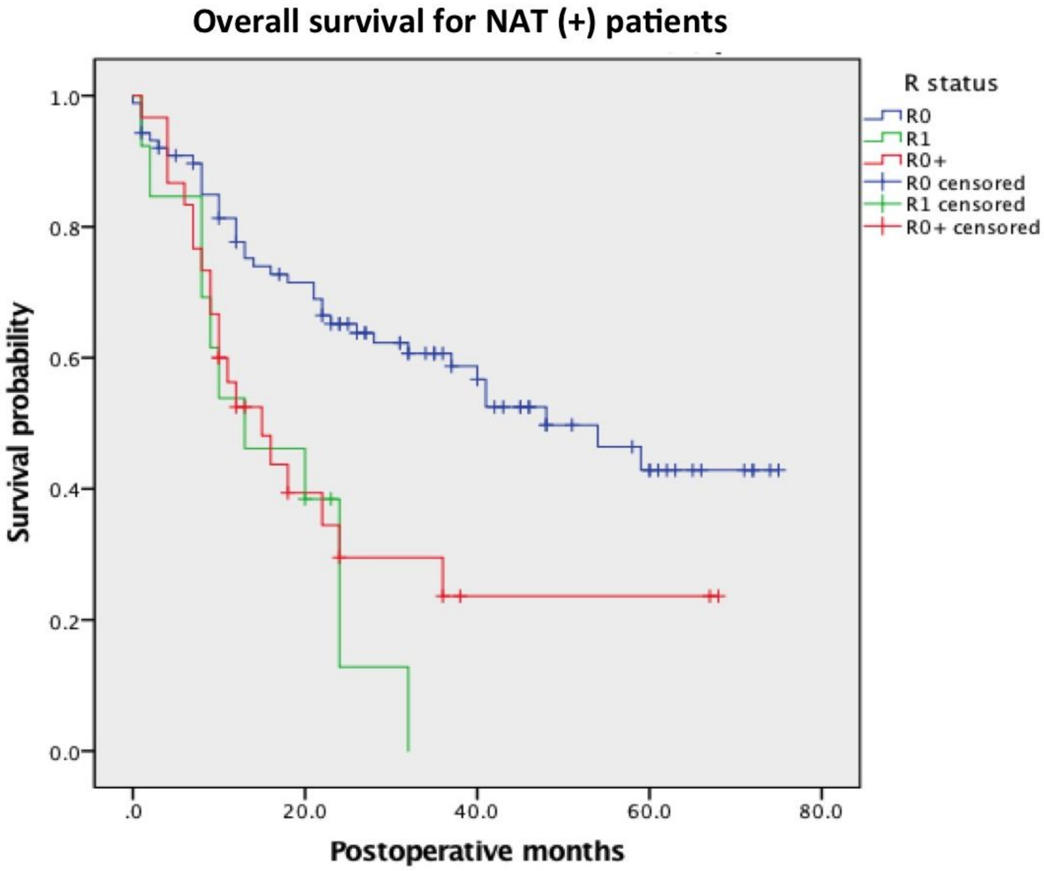

Nb at risk	0 mo	20 mo	40 mo	60 mo	80 mo
R0	87	57	27	11	0
R0+	29	8	1	0	0
R1	12	4	0	0	0

Figure legend: R0 patients had a median OS of 48 months (95%CI 30.9-65.1), significantly better than R0+ (median 15 months 95%CI 7.5-22.4, p=0.002) and R1 patients (median 13 months, 95%CI 0.1-25.9, p<0.001). The difference was not significant between R0+ and R1 patients (p=0. 461).

R0: resection margins >1mm R0+: resection margins ≤1mm R1: resection margins 0mm

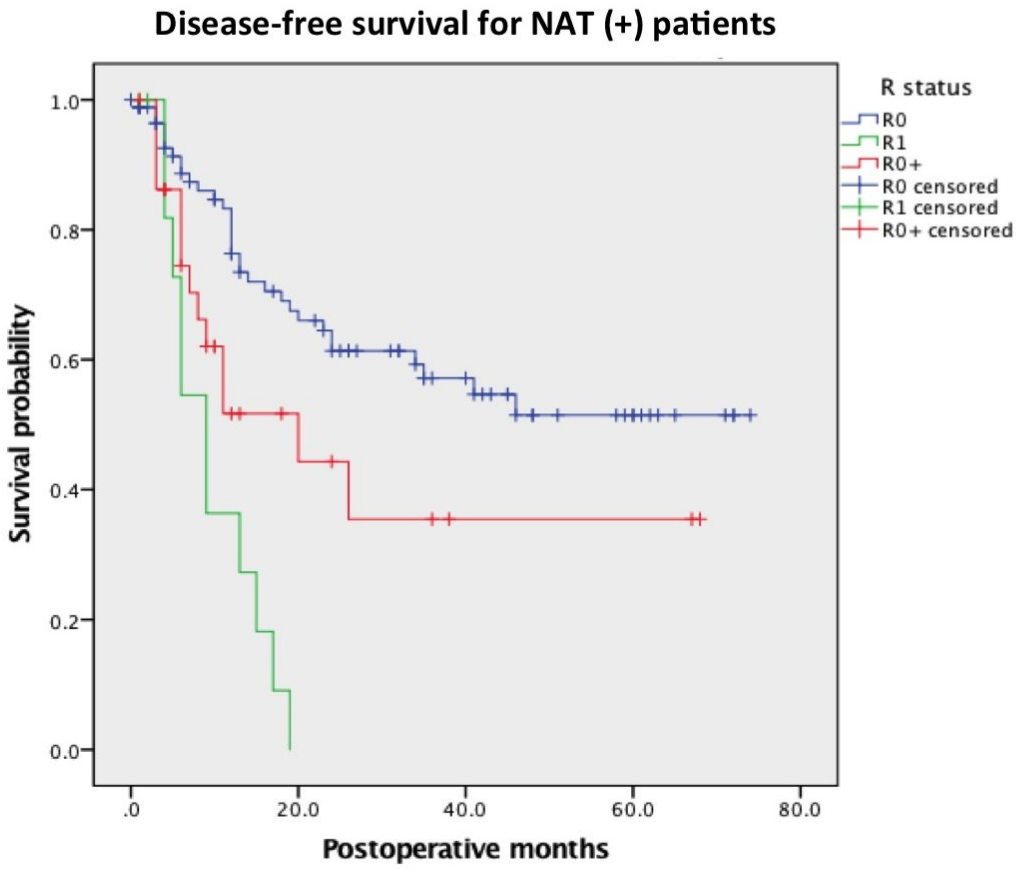

Nb at risk	0 mo	20 mo	40 mo	60 mo	80 mo
R0	86	44	23	8	0
R0+	29	6	1	0	0
R1	12	0	0	0	0

Review

Figure legend: R0 patients had a mean DFS of 46.6 months (95%CI 39.4-53.7), significantly better then R0+ (median 20 months 95%CI 26.3-33.7, $p=0.032$) and R1 patients (median 9 months, 95%CI 5.9-12.1, $p<0.001$). R0+ patients had a significantly better survival than R1 patients ($p=0.015$).

R0: resection margins >1mm R0+: resection margins ≤1mm R1: resection margins 0mm
